# Conflicts of interest for members of the US 2020 dietary guidelines advisory committee

**DOI:** 10.1017/S1368980022000672

**Published:** 2022-03-21

**Authors:** Mélissa Mialon, Paulo Matos Serodio, Eric Crosbie, Nina Teicholz, Ashka Naik, Angela Carriedo

**Affiliations:** 1 Trinity Business School, Trinity College Dublin, Dublin, Ireland; 2 ISER, University of Essex, Wivenhoe Park, CO43SQ Colchester, United Kingdom; 3 School of Community Health Sciences, University of Nevada, Reno, NV, USA; 4 Ozmen Institute for Global Studies, University of Nevada, Reno, NV, USA; 5 The Nutrition Coalition, New York, NY, USA; 6 Corporate Accountability, Boston, MA, USA; 7 World Public Health and Nutrition Association, London, United Kingdom; 8 Department for Health, University of Bath, Bath, United Kingdom

**Keywords:** Dietary guidelines, Conflicts of interest, Commercial determinants of health

## Abstract

**Objectives:**

To measure incidence of conflicts of interest (COI) with food and pharmaceutical industry actors on the advisory committee for the 2020–2025 US Dietary Guidelines for Americans (DGA) and assess the adequacy of current mechanisms to disclose and manage COI among the committee’s members.

**Design:**

We compiled longitudinal data from archival sources on connections between members of the DGA’s advisory committee and actors. We hypothesised that these committee members, who oversee the science for the most influential dietary policy in the USA, might have significant COI that would be relevant to their decision making. Disclosure of COI on this committee was recommended in 2017 by the National Academies of Sciences in order to increase transparency and manage bias, but public disclosure of the committee’s COI does not appear to have taken place.

**Setting:**

The committee was composed of twenty experts.

**Participants:**

None.

**Results:**

Our analysis found that 95 % of the committee members had COI with the food and/or pharmaceutical industries and that particular actors, including Kellogg, Abbott, Kraft, Mead Johnson, General Mills, Dannon and the International Life Sciences, had connections with multiple members. Research funding and membership of an advisory/executive board jointly accounted for more than 60 % of the total number of COI documented.

**Conclusions:**

Trustworthy dietary guidelines result from a transparent, objective and science-based, process. Our analysis has shown that the significant and widespread COI on the committee prevent the DGA from achieving the recommended standard for transparency without mechanisms in place to make this information publicly available.

In December 2020, the ninth version of the Dietary Guidelines for Americans (DGA) was released^(1)^. Since being introduced in 1980, the DGA has been revised every 5 years and is meant to provide dietary advice to ‘meet nutrient needs, promote health and prevent disease’^(2)^. In the USA, the DGA are required by statute^(3)^ to form the foundation for all national nutrition programmes (which are amounting to nearly $100 billion/year^(4)^) and guide states and local governments, healthcare professional training, hospitals and community groups, amongst others, as overarching dietary recommendations^(5,6)^.

For the development of the 2020–2025 DGA, there was a three-step process in which the US Departments of Agriculture (USDA) and Health and Human Services (HHS): (1) identified nutrition topics to be reviewed, including through a public consultation; (2) appointed an external Dietary Guidelines Advisory Committee (DGAC), composed of twenty experts from the fields of nutrition and medicine, to review the scientific evidence for these topics and (3) and wrote the DGA based largely on the DGAC’s scientific report^(1)^.

The DGA recommendations are important since they are meant to shape what Americans eat and drink. In particular, the food industry has historically been observed to seek to influence the DGA process in its favour, for example by pushing for recommendations for particular foods or food groups, such as dairy products, grains or meat^(7)^. For instance, of the comments submitted by organisations to the public consultation for selection of topics for the development of the 2020–2025 DGA (step 1, described above), nearly 70 % were from industry actors, particularly those in the food industry^(8)^. Moreover, trade associations such as the American Beverage Association, the Grocery Manufacturers Association (now the Consumer Brands Association) and the National Potato Council, as well as companies like Unilever, nominated experts to be appointed to the DGAC through an informal step, prior to step 2 of the process described above^(8,9)^.

The scientific report produced by the DGAC might also have been subject to influence, for example, if DGAC members had financial conflicts of interest (COI) and existing relationships with industry actors. COI among DGAC members is therefore a key question in the development of the DGA. DGAC members are considered temporary workers for the federal agencies and are therefore required to follow the USDA ethics rules, to ‘place loyalty to the United States Constitution, Federal laws and ethical principles above private gain’ and ‘may not “participate personally and substantially” in a “particular matter” in which (they) have a financial interest’^(10)^ (where financial interest also includes an ‘imputed’ financial interests, which are those involving spouses, children and organisations). COIs (financial and non-financial) of nominees for the DGAC were therefore considered by the USDA Office of the Secretary and reviewed by the USDA Office of Ethics before DGAC members were appointed^(11)^. Once appointed, DGAC members were then required to report their financial interests (hereafter referred to as ‘COI’) through a US Office of Government Ethics Form 450^(12)^ (these include assets, sources of income, debt, outside positions, gifts and travel reimbursements). Upon reviewing these materials, the USDA ethics officials in charge of the process stated that ‘none of the 20 committee members reported any entries (…) that would prevent them from being appointed and providing the complete range of duties required of a Dietary Guidelines Advisory Committee member’^(11)^. The documents used for that screening process and all COI reported/disclosed by the DGAC members annually thereafter during their term on the committee were meant to be posted on DietaryGuidelines.gov, the official website of the DGA, as indicated in the DGAC scientific report (Part C methodology^(11)^). However, those COI reports could not be found online at the time of our data collection, limiting the possibility for public scrutiny of the COI of DGAC members.

Additionally, in 2017, the National Academies of Sciences, Engineering, and Medicine (NASEM) produced a Congressionally mandated report on the DGA process in which the Academies issued a four-part recommendation ‘to enhance transparency, manage biases and COI to promote independent decision making’^(13)^. Specifically, the NASEM recommended that USDA-HHS should disclose how provisional DGAC nominees’ biases and COI are identified and managed, by, among other things, ‘creating and publicly posting a policy and form to explicitly disclose financial and nonfinancial biases and conflicts’^(13)^. This recommendation reflects the now-common practice, for example of academic journals, to require public COI disclosure for scientific experts^(14)^. This is also aligned with the standards set by the US Institute of Medicine, that when developing clinical practice guidelines, individuals ‘who have a conflict of interest should not represent more than a minority of the group’^(15)^. The 2020 DGAC scientific report addresses the NASEM’s recommendation to manage COI and biases, yet its discussion is highly generalised, without discussing any specific COI.

In the present study, we therefore aimed to document the COI of DGAC members (in their capacity as scientific experts), and in particular their relationships with industry actors, since these relationships could directly have affected the committee process and decisions^(7)^. Our goal for this study was to bring to light the COI of DGAC members, which we consider to be vital information as a backdrop to a critical and informed assessment of the DGAC scientific report. We also comment on the lack of mechanisms currently in place at the USDA-HHS to publicise information pertaining to COI among its DGAC members.

## Methods

We conducted searches in January and February 2021, using publicly available data. Searches were led by PMS, MM and AC. Data were managed on Excel 2010. The list of DGAC members is available in Appendix F-3 of the Scientific Report of the 2020 Dietary Guidelines Advisory Committee^(11)^.

### Data on conflicts of interest

For this study, we defined COI as relationships between a DGAC member and an industry actor in a given year. We documented the year in which the COI was disclosed as the year for which the COI existed, even if the relationship between the DGAC members and the organisation might have been maintained for a longer period of time than that disclosed. For example, a 5-year research grant yielding one published paper was only considered once as a COI (for the publication of the article), given that we lacked evidence for the whole 5-year period. This drawback is only avoidable for those cases where the duration of the COI was disclosed (e.g. start and end date of the grant). Furthermore, lacking evidence to the contrary, we considered funding from industry to be a COI for any DGAC member who is a co-author on a study sponsored by industry. And on the contrary, if the relationship or grant was mentioned in multiple publications in the same year, we counted it once. This approach does not distinguish between cases where a DGAC member might have received more than one grant in the same year from the same industry actor, as we count that as a single instance of a COI.

We argue that the time dimension is important in order to shed light on long-term relationships between industry and DGAC members. Therefore, we considered COI without date restrictions, allowing us to go as far back in time as information is publicly available.

We took a conservative approach using exclusively primary data to obtain evidence of a COI. We considered primary data sources as those platforms where information about COI is disclosed either directly by a DGAC member (e.g. scientific publication or a Curriculum Vitae) or by the institutions to which they were affiliated (e.g. bios on institutional websites). Primary data sources were excluded where a COI was discussed without a reference to the original information source.

We focused on the COI of DGAC members with corporate actors from the food, drink, and pharmaceutical industries, as well as third parties working with them such as trade associations or front groups. We included pharmaceutical companies because some sell infant nutrition products and often offer devices or drugs that compete with food-based solutions to chronic diseases. We searched for information specifically on the DGAC members, not their families or other third parties, as included in Form 450.

Below, we expand on the iterative process through which COI were identified, collected and documented.

### Sources of information and data collection

First, we retrieved the scientific publications of DGAC members from the Web of Science Core Collection (WoS CC). In those publications, we searched for evidence of COI in three different sections: (1) institutions to which the DGAC members were affiliated; (2) funding acknowledgments sections and (3) declarations of interest sections. Second, we conducted snowball searches, using the personal and institutional websites of all DGAC members, which often provided us access to a Curriculum Vitae that allowed us to both triangulate the data retrieved from WoS and to obtain more detailed information. We then searched other webpages identified through these sources. We conducted additional searches on Google, using the names of the members as search terms, to uncover other COI. We also reviewed information from the media and civil society organisations mentioning the COI of the DGAC members for the 2020–2025 DGA^(8,15–19)^ (albeit this list is not comprehensive). Using that information, we searched for primary sources when a COI was mentioned. All the COI compiled in our database were independently verified by PMS, MM and AC.

We grouped relationships between DGAC members and industry actors in the following categories: ‘research funding’; ‘editor’ of a publication run by an industry actor; ‘speaker/honoraria’ for paid participation in ‘sponsored events’ or for participation in or organisation of industry funded conferences; ‘board/committee member’ (hereafter ‘board member’) if serving on an advisory/scientific committee or board of directors or as a liaison between industry and another organisation, ‘employee,’ ‘award/prizes’ and ‘consultant.’ The classification scheme was designed by PMS, MM and AC, with input from the other authors, and each relationship was classified independently by the three authors. Inter-coder reliability was greater than 0·9, with new coding being jointly discussed and agreed upon for the discrepancies in classifications.

In addition, we took note of the public research funding received by DGAC members from the USDA-HHS, using the grants search engines for these federal agencies and information on any executive position a DGAC member might have held in those agencies. This process allowed us to compare the extent of the COI of the DGAC members against their work that was supported with public funding. We compared career-long ties to industry actors against the support received from federal agencies, mostly in the form of competitively awarded research grants. We believe that both pieces of information are important for the audiences targeted by the DGAC scientific report and DGA guidelines, allowing those audiences to be more informed to critically digest the recommendations and to put both elements into perspective.

### Data analysis

The relationships documented between DGAC members and industry actors were analysed through the lenses of network and sequence analyses. To better grasp the composition of industry actors that cultivated relationships with DGAC members, we constructed three network plots: (1) a bipartite, valued network graph depicting the links between DGAC members and the top fourteen industry actors with most connections to DGAC members; (2) an bipartite, valued network graph depicting links between industry actors and the top six DGAC members with most ties to industry; (3) an bipartite, valued network graph depicting links between DGAC members and industry actors with ties to, at least, two DGAC members and (4) one-mode projection network graph linking industry actors based on whether they were connected to the same DGAC members.

The network plots treat the data as a pooled data, disregarding the longitudinal nature of the relationships. To further explore the time dimension, we used sequence index plots that summarise the relationships with industry for each DGAC member throughout her or his career. The original data spreadsheet, available in the replication materials, stores both the COI as available in the primary source, along with a coding scheme we created in order to distill the different relationships between researchers and industry actors into categories we could analyse.

## Results

We found that nineteen out of the twenty DGAC members had some form of relationship with industry actors (only one person had no COI). In Table 1, we report the instances of COI we identified.

**Table 1:** Frequency table for type of conflicts of interest (COI) for all Dietary Guidelines Advisory Committee (DGAC) members

COI category	# of instances of COI	% of overall COIs
award	35	4.9%
board member	155	21.5%
consultant	105	14.6%
editor	6	0.8%
employee	20	2.8%
research funding	287	39.9%
speaker/honoraria	112	15.6%
	720	

Research funding and membership of an advisory/executive board jointly accounted for more than 60 % of the total number of COI documented. The percentages can be explained in part by our approach in quantifying instances of COI, as we counted a COI anew for each year it was disclosed. Therefore, a research grant that was awarded for 5 years may be counted as five separate instances of a COI. Nonetheless, given that our method is also prone to underreporting, these percentages illustrate what are effectively the two main pillars underpinning long-term relationships between scientific experts and industry actors: (1) funding for research projects and (2) advisory roles in corporate boards. In both cases, there seems to be an interplay between the strategic interests of industry actors, the professional interests of the researcher and, ultimately, the scientific work produced by the former.

**Fig. 1:**
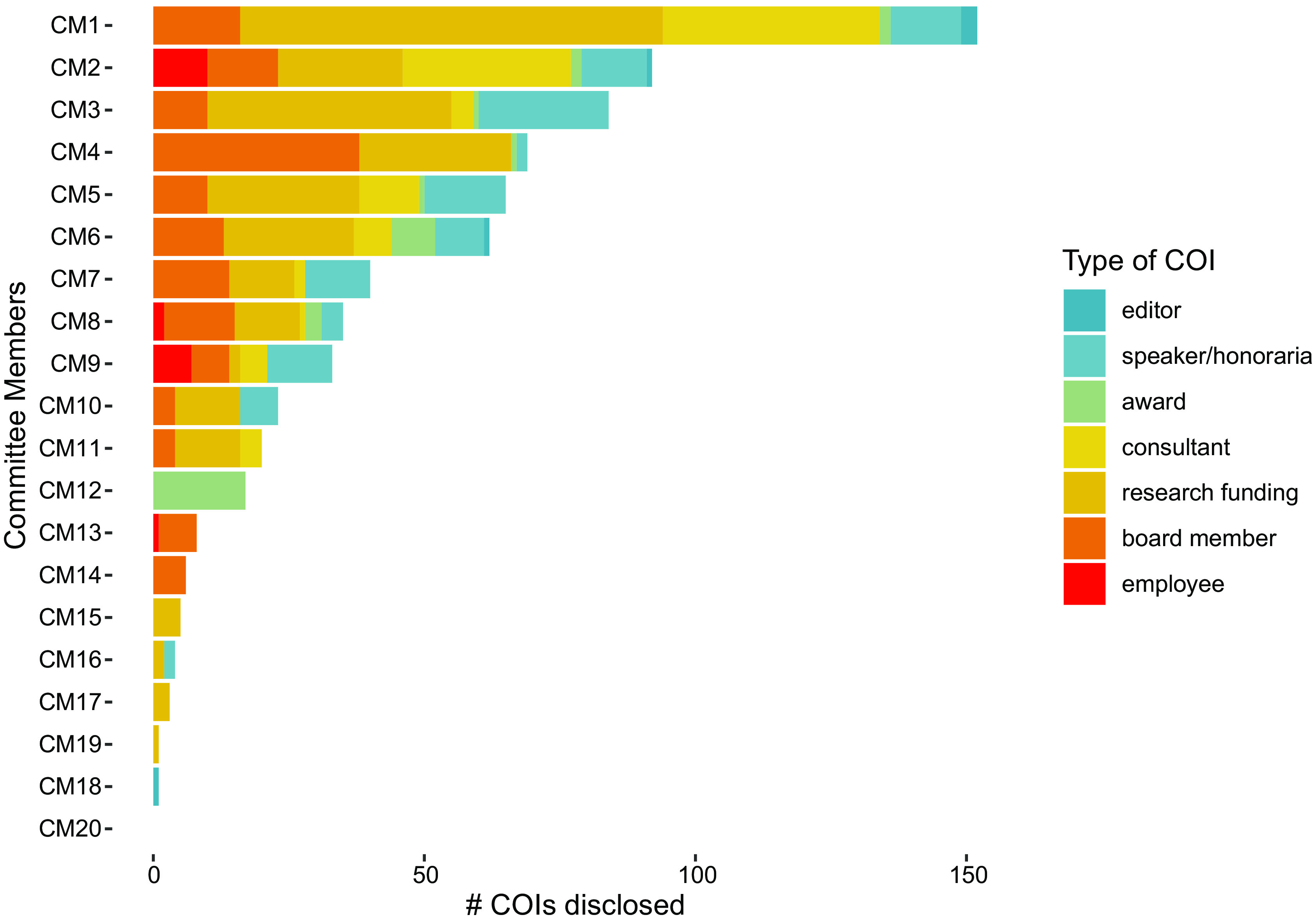
Number of conflicts of interest (COI) by type, for each Dietary Guidelines Advisory Committee (DGAC) member

In Fig. 1, we break down the instances of COI for each DGAC member over time. This is particularly relevant when considering that the recommendations made by the DGAC would arguably have a direct impact on the revenues of the industry actors that had already an established relationship with its own members. In addition, most of the DGAC members had repeated instances of COI of different types (per our classification scheme), and in some cases developed long-term relationships with industry actors that have spanned almost their entire careers.

We also analysed the composition and organisation of the network of industry actors and their relationships with DGAC members. In light of the large number of industry actors disclosed in COI statements by DGAC members (*n* 129), we zoomed in on the main networks and focused the analysis on sub-graphs, in order to highlight the main features of interest for the purposes of this paper. The full network connecting industry actors to DGAC members can be found in Appendix 1, and an interactive version of this network can be found in the web appendix.

**Fig. 2:**
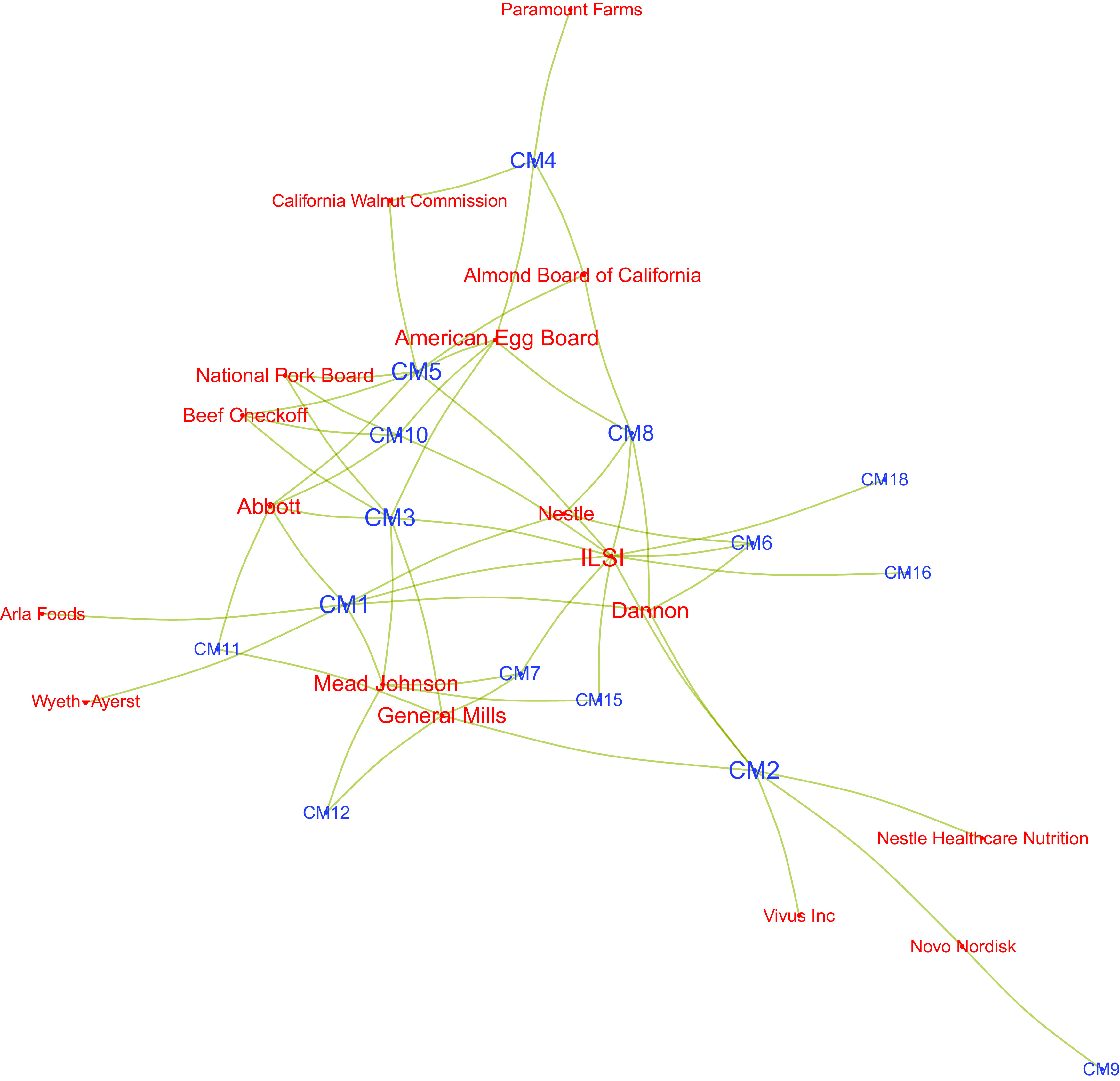
Network of the fourteen industry actors with the most connections to Dietary Guidelines Advisory Committee (DGAC) members. Ties represent conflicts of interest (COI) involving both actors

Figure 2 restricts the network to the top fourteen industry actors, that is, those which appear in more than twelve instances of COI in our data. Although this network reveals some degree of core/periphery structure, with the International Life Science Institute (ILSI), funded by food industry actors^(20)^, as well as Dannon, and Abbott, clearly in the centre of the network, few of the industry actors depicted having ties to only one DGAC member. This suggests the patterns of connections amongst these top fourteen industry actors are not long-term relationships with one DGAC member but, rather, are characterised by multiple relationships (of unclear duration) with several DGAC members. From the perspective of conveying the industry’s connections to the committee, a corporation tied to several DGAC members is in a better position of having its interests represented in the Committee. Table 2 examines this issue further and provides a breakdown of COI for the top fifteen industry actors, while Table 3 provides a similar breakdown for DGAC members.

**Table 2:** Top fifteen industry actors by overall number of conflicts of interest (COI) along with the number of unique Dietary Guidelines Advisory Committee (DGAC) members with whom the industry actors have had a relationship

industry actor	# of instances of COI	# of DGAC members
ILSI	45	11
Mead Johnson	43	5
California Walnut Commission	33	2
Dannon	30	4
General Mills	29	5
Nestle Healthcare Nutrition	25	1
Abbott	22	5
American Egg Board	20	5
Novo Nordisk	19	2
Vivus Inc	17	1
Beef Checkoff	16	3
Nestle	16	3
Wyeth-Ayerst	16	1
Arla Foods	15	1
National Pork Board	15	3

**Table 3:** Dietary Guidelines Advisory Committee (DGAC) members ranked by overall number of conflicts of interest (COI), along with the number of unique industry actors with whom each DGAC member had a relationship

DGAC member	Overall # of COI	# of industry actors connected to
CM1	152	31
CM2	92	12
CM3	84	31
CM4	69	17
CM5	65	32
CM6	62	22
CM7	40	15
CM8	35	13
CM9	33	10
CM10	23	9
CM11	20	6
CM12	17	2
CM13	8	2
CM14	6	2
CM15	5	4
CM16	4	3
CM17	3	2
CM19	1	1
CM18	1	1
CM20	0	0

**Fig. 3:**
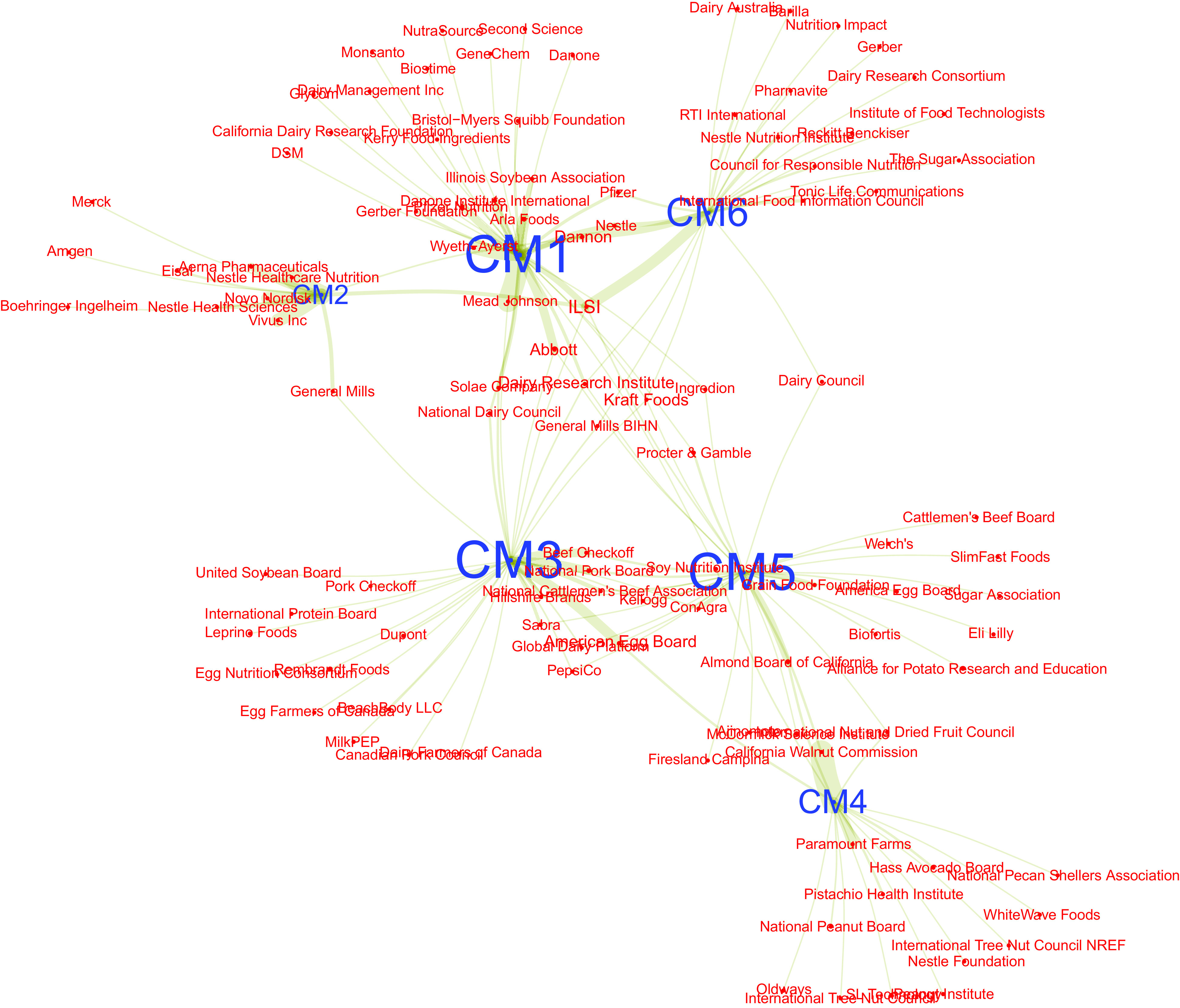
Induced network of six Dietary Guidelines Advisory Committee (DGAC) members with the most ties to industry. Each tie represents a conflicts of interest (COI) involving a member of the committee and a corporation

Shifting our focus from the industry actors to DGAC members, in Fig. 3, we plot the combined individual networks of the six DGAC members with the most ties to industry. Akin to what we observed before, several global industry actors appear as bridges amongst several DGAC members, although the majority of industry actors are tied to only one of the top six DGAC members. Although this pattern could be a function of available resources, with larger companies investing in relationships with multiple researchers, it may also illustrate a strategy by corporations to develop relationships that maximise their impact on science and policy.

By the same token, DGAC members appear to disclose relationships each with a different group of industry actors, which are largely a reflection of how their own research speaks to a different industry sector, albeit most of them exhibit ties to corporations both in the food and pharmaceutical sectors.

We provide an industry sector breakdown in Table 2, which lists the top fifteen industry actors with the most connections to DGAC members over time, while also showing the number of unique DGAC members to which they have ties. Table 3 provides a similar list, but is focused on DGAC members.

**Fig. 4:**
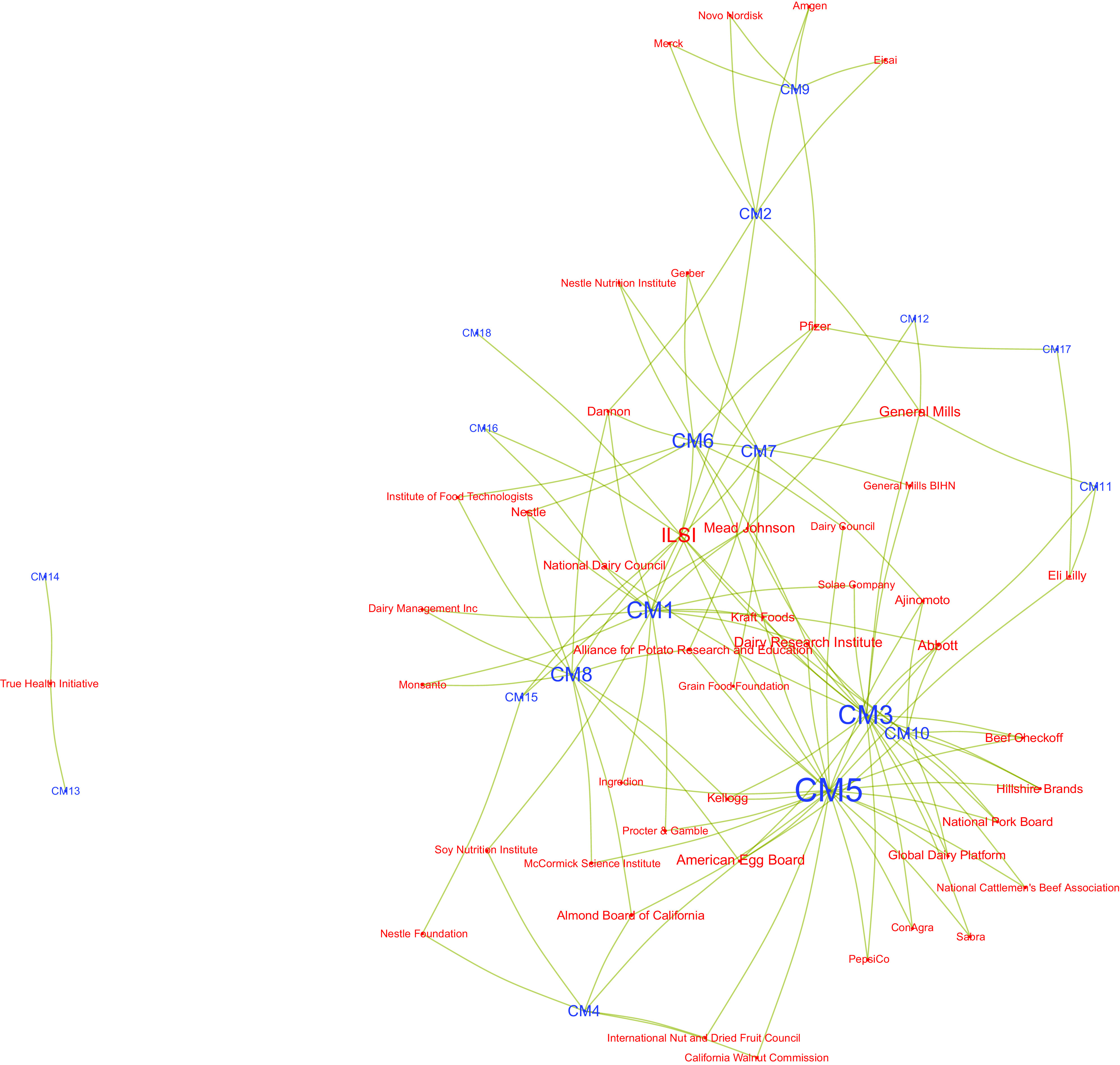
Network plot depicting relationships between industry actors and Dietary Guidelines Advisory Committee (DGAC) members, for those industry actors who had ties to at least two DGAC members. Node labels are colored by type of actor (blue are DGAC members; red are industry actors)

**Fig. 5:**
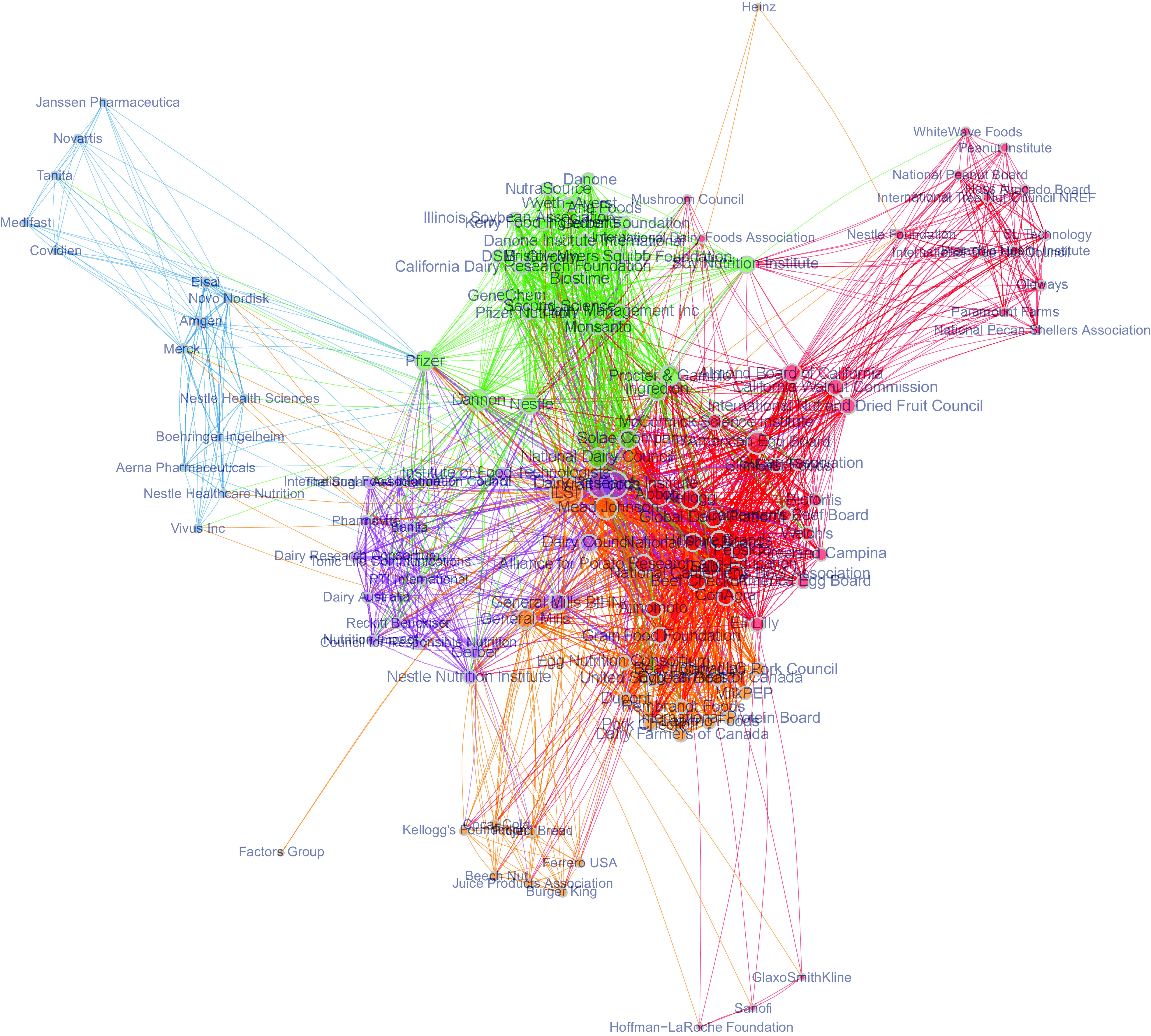
Bipartite projection, one-mode, undirected, valued graph, depicting number of shared

ILSI had the greatest number of ties over time with the largest number of DGAC members (also illustrated below, in Figs. 4 and 5). Other actors also show a different set of connections in different ways: for example, the California Walnut Commission was listed thirty-three times in COI declarations but was tied to only two DGAC members. The same analysis can be applied to DGAC members individually: some have a long list of COI, whilst others have had fewer COI, yet with a greater number of industry actors. These are the same dynamics exemplified in Fig. 2.

If we expand Fig. 2 to include all industry actors tied to at least two DGAC members, the network loses its core-periphery structure while still revealing the industry giants at the network’s centre. That way, we can observe that Kellogg, Abbott, Kraft, Mead Johnson, ILSI, General Mills and Dannon are well positioned to advance their interests within the DGAC given the existence of relationships (in some cases long-held) with several DGAC members. Although these networks reveal an array of avenues through which DGAC members may be influenced by industry, it is also possible that they are unable to consider the specific interests of each and every one of the actors with whom they have a COI, not only because some of those relationships are no longer active but also due to the sheer number of actors involved.

In Fig. 5, we link industry actors based on whether they were identified in COI involving the same DGAC members. The network links industry actors that developed relationships with the same DGAC members. The nodes in the network are grouped and coloured by the Louvain community search algorithm, which partitions the network into several communities, that is, subgroups which are only loosely connected to the main network. These communities are prominent in the network, and we could argue that they represent different sectors of the industry. Therefore, even though the sheer scale of COI involving different industry actors may preclude a DGAC member from acknowledging each individual interest in drafting the DGAC report, there is surely an element of sector-wide influence that could make DGAC members vulnerable to representing the interests of industry sectors. This situation could result when an industry altogether establishes relationships with multiple DGAC members.

**Fig. 6:**
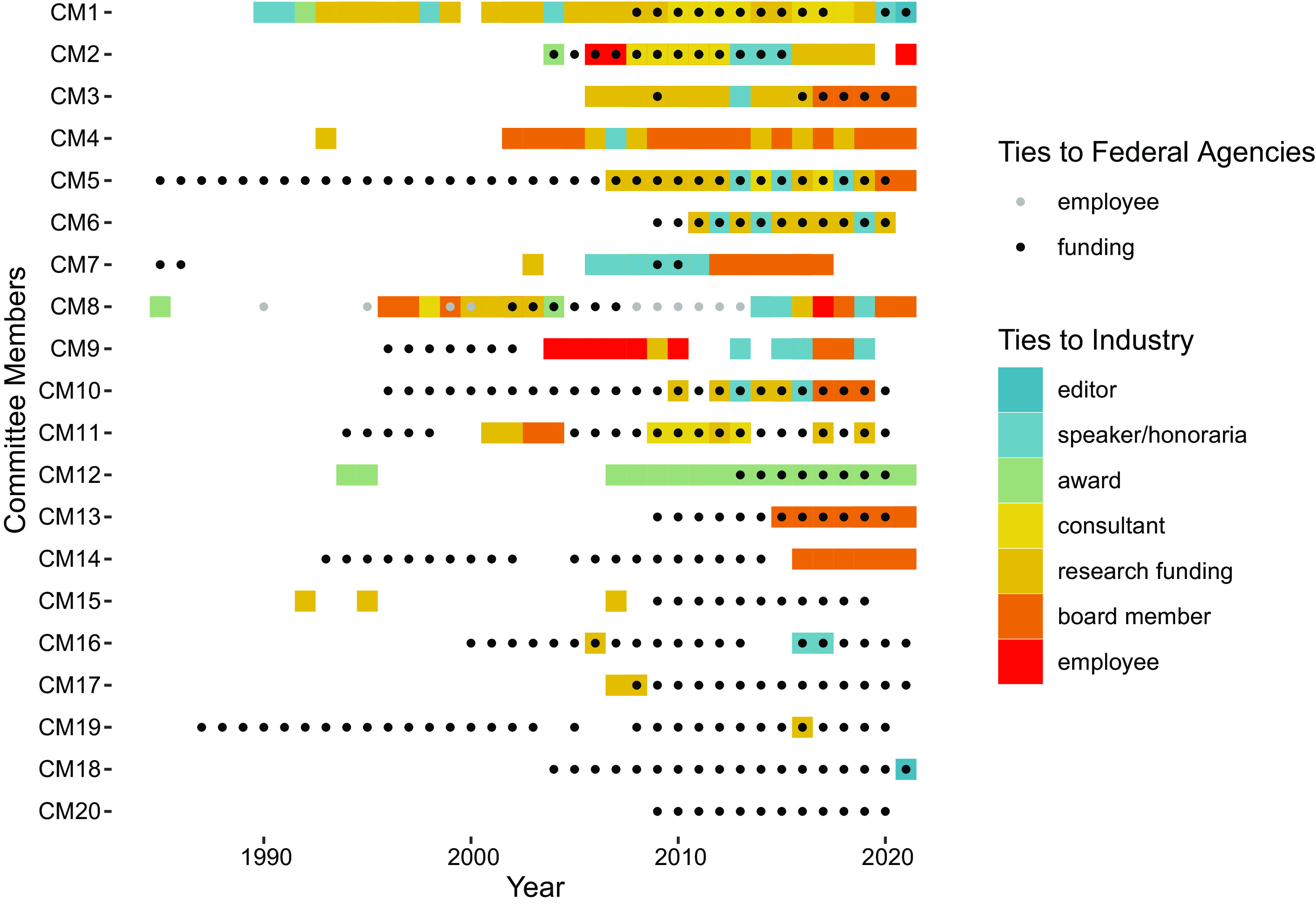
Dietary Guidelines Advisory Committee (DGAC) members ties with the industry and ties to the USDA-HHS. Black dots denote active grants from federal agencies. Gray dots denote employment with federal agencies

Finally, we considered the research grants that DGAC members received from federal agencies through competitive processes and any existing executive position that members may have held in those agencies. In Fig. 6, we use a sequence index plot to map in time the occurrence of COI, by type and overlay any ties with the public sector (by type), for each DGAC members.

With the exception of a few cases, DGAC members were typically successful at obtaining research funds from governmental agencies, and these ties are usually of longer duration than their ties to industry. This has to be put in perspective and compared with their relationships with industry.

## Discussion

Our results show that nineteen of the twenty DGAC members (95 %) had at least one tie to an industry actor. In the DGAC, a majority of members had more than twenty interactions with industry actors and interacted with more than ten industry actors each. The most prevalent type of COI was research funding, followed by DGAC members being on a board/committee in a company, and consultant positions. Some industry actors, such as Mead Johnson, General Mills and Kellogg’s, and the industry-funded organisation, ILSI, have interacted with an extensive number of DGAC members. Conversely, other industry actors have prolonged relationships with only a handful of DGAC members. Amongst the top fifteen industry actors by overall number of COI are ILSI and three trade associations or programmes funded by them (California Walnut Commission, Almond Board of California and Beef Checkoff). Each of these actors has diverse means and ends to potentially influence scientific research and the DGA process, although it is beyond the scope of this paper to research instances of this potential influence on outcomes.

We observed the existence of extensive, varied and long-standing relationships between some DGAC members and industry actors whose products are directly affected by the DGAC report´s recommendations as well as the DGA themselves. Systematically mapping the COI of DGAC members against the evidence discussed in the DGAC scientific report was beyond the scope of our study. However, we noted some examples where DGAC members had COI that could have affected how those members perceived or evaluated evidence in relation to particular foods, as documented elsewhere^(7)^. We acknowledge the dangers such financial COI might have had on the outcomes. For example, the Pregnancy and Lactation Subcommittee of the DGAC had six members, four of whom, or two-thirds, had COI involving manufacturers of breastmilk substitutes: CM1, CM7, CM15, CM4 all had instances of COI with Mead Johnson and CM1 had COI with Wyeth and Abbot. The Birth to Age 24 Months Subcommittee, which also addressed infant and young child nutrition, had four of its six members having COI involving manufacturers of breastmilk substitutes: CM1, CM7, CM15 also served on the Pregnancy and Lactation Subcommittee, with the same COI mentioned above, and CM10 had at least one COI with Abbott^(19)^. There is evidence that those companies producing breastmilk substitutes regularly use science and try to influence policy in order to protect and promote the sales of their products, and their relationships with DGAC members may have had a direct impact on the work of those members^(21)^. This pattern is certainly not unique in the food industry^(22–24)^. It is but one example of the potential impact COIs may have had on outcomes in the DGA process. It is beyond the scope of this paper to analyse fully all the potential such impacts of so many corporate and industry actors.

By looking at a researcher’s entire career (albeit limited by data constraints, such as indexing of funding metadata in bibliometric databases), we were able to identify different COI which, in analyses of shorter duration, would have instead been given equal weight. Consider the case of researcher A, who received a grant from an industry actor the year before sitting on the DGAC for the first time in her career, and researcher B, who had worked on and off with industry for a period of 20 years, but who happened to have no active grants in the year prior to being appointed to the DGAC. Putting a researcher’s involvement with industry in perspective over his or her entire career might change that assessment.

We noted that, with the exception of a few cases, DGAC members were typically successful at drawing research funds from governmental agencies and had the possibility to get funding from other sources than from corporations. Lack of funding might therefore not be the best explanation for working with corporations. The reasons for top-national experts to work with industries merit further investigations.

Importantly, based on the information we collected, we could not say whether a COI had led to bias, as this was beyond the scope of this article. Nor we did study the process for developing the DGAC scientific report and how COI and other forms of influence by industry actors may have impacted its writing; this could be the subject of future analyses. Moreover, this was not a study of the influence of industry actors over the development of the DGA themselves, which is discussed elsewhere^(7)^. For instance, it has been documented that industry actors directly nominated individuals on the DGAC, as discussed earlier^(18)^. Similarly, we did not study the COI of the USDA-HSS employees who were involved in preparing the DGA. These topics will merit additional searches^(16,9,25)^. The use of what is called a ‘revolving door’ might also be problematic, with, for example, the Secretary of Agriculture, who spent much of his career in the agribusiness sector, having the ultimate say over the final content of the guidelines^(26)^. Finally, in 2020, there were members of the Congress, some of whom received donations from the alcohol industry, who questioned the science on alcohol discussed in the DGAC scientific report, with the USDA-HHS overriding the recommendation on restricting alcohol consumption in the DGA^(27)^. Thus, the influence of industry actors extends well beyond individual COI and the process for developing the scientific report that served as a basis for the writing of the DGA.

However, it is well known that industry funding and COI have a negative impact on both research results and the research agenda^(22–24)^. Our findings here are particularly worrisome, as industry influence and COI can result in diverting the scientific process underpinning the US national dietary guidelines, to one that is responsive to profit-driven interests rather than the public health^(22)^. It is critical to underscore the DGA’s impact on public health, especially for communities who are most impacted by diet-related diseases. For Americans to be able to trust the guidance from the DGA as sound, objective and science-based, it is imperative to ensure that each step of the process, from the selection and appointment of the DGAC to the final release of the DGA, is publicly accessible, transparently administered and largely free of COI and influence from actors whose profit-driven interests are often at odds with those in public health. Our analysis of COI of DGAC members has shown that this is far from true.

Current mechanisms used by the USDA-HHS to assess the COI of DGAC members may have limitations, which could have direct implications on the DGAC’s recommendations that guide the DGA. The current process for assessing COI, based on annually self-reported disclosures, does not capture the long-standing relationships between the DGAC and industry actors that we identified here. A ‘COI timespan’ of at least 3–5 years is normal, although our paper demonstrates that a longer timespan would be beneficial to understanding the breadth and depth of an expert’s long-term relationships with industry. Moreover, to be as thorough as possible, COI declarations should include past positions, revolving door situations and COI involving third parties, such as industry front groups (e.g. ILSI). Funding being only one type of COI, with membership of a board/committee or consulting being also central in our findings, these other forms of relationships need to be addressed in the context of the prevention and management of COI. We do not know if these were included by the USDA-HHS in their COI review process.

There is, in addition, a need for more transparency in the process for selecting DGAC members – a process where all pertinent information is made public (e.g. information contained in Form 450). The DGAC report states that Forms 450 were posted online, but we could not find them on the DGA website at the time of our data collection. It is this paper’s contention that the USDA-HHS should publicly post all COI of appointed DGAC members, as recommended by the 2017 NASEM report permanently during and after completion of the DGA. As also recommended by NASEM, these COI should be managed throughout the DGA process. Ideally, transparency and management of COI should also be applied to all USDA-HHS employees involved in the DGA process, since ultimately, these employees are entirely responsible for writing the final DGA policy. The DGAC expert report is, in fact, just one input to that final policy. Currently, the writing process by USDA-HHS officials is not managed transparently. Mechanisms to increase transparency and manage COI should, in our view, also address these USDA-HHS employees and should be accompanied by the participation of experts on COI and ethics.

### Limitations

Our study has some limitations. While quantifying COI is one of the main challenges we faced in this paper, it is also one of the main contributions we hope to offer the research community as a whole. The starting point must be the recognition that publicly available information is severely limited, heterogeneous at best and inconsistent at worst. We worked through these hurdles to produce a uniform database that documents instances of COI/year.

The method used to uncover financial COI has important caveats, which can lead to misreporting DGAC members’ relationships to industry (both over and under). COI statements can be vague in nature, often using expressions such as ‘consultancy, honoraria or speaking engagements’, which prevents us from accurately describing the relationship. In the case of COI related to research funding, and in the absence of details on the research grant that originates the funding relationship between a DGAC member and an industry actor, our strategy was prone to underreport the length of the relationship. For example, if a DGAC member was awarded a 5-year research grant that only yields one publication, in which the funding was disclosed without further details, we log this relationship as taking place in the year of publication, therefore we might have underestimated its length. Given that we documented COI by year, we were unable to consider any disclosed tie for which a time period was missing. For example, this is often the case in brief bios available on the DGAC members’ institutional webpages, which disclosed relationships with numerous industry actors without mentioning the time period during which those relationships took place. In addition, peer-reviewed publications only provide self-reported COI, so we might have underestimated the number and length of interactions. We triangulated these data with other available public data, but information was scarce. We assumed that a tie with a particular industry happened in the year disclosed/recorded, but in the case of consultancies or funding for research, these ties might have lasted several years, as it was not clearly disclosed/recorded as such in the publicly available information we used.

In addition, we relied on availability of data from bibliometric databases, which have only recently included funding and COI disclosures as searchable metadata in their databases since 2008. We could not collect information on COI in publications before that year. Despite our efforts to replicate previous publications on COI of the 2020 DGAC, many sources used in those articles were no longer publicly available at the time of our data collection, which in part accounts for the discrepancies between those earlier findings and ours. Furthermore, since we relied on self-reported COI statements in those publications, we lack objective and detailed information on the nature of the relationship between researchers and industry: for instance, seldom are both the start and end dates for a research grant from an industry actor made available. In light of the obstacles, our conservative approach opted for leaving out relevant data given our inability to place it in a moment in time or given our inability to characterise it fully. Further research on each of the peer-reviewed documents obtained or the inclusion of other methods might have been applied but were out of the scope of this study.

## Conclusion

Our findings suggest that the vast majority of DGAC members had at least several COI directly relevant to their work on the scientific report that underpins the 2020–2025 DGA. Some DGAC members were found to have maintained prolonged relationships with industry actors in the food and pharmaceutical industries, both of which have a direct interest in DGA recommendations. The current ethics process of the USDA-HHS for assessing COI of DGAC is based on self-reported disclosures that are not made available to the public. This practice is contrary to the recommendation of the 2017 NASEM report and to general practice in the fields of nutrition and medicine. Existing COI for more than a minority of the DGAC is also contrary to the standard set by the Institute of Medicine. A robust examination of COI for all DGAC members, in addition to the management of these potential biases, would be consistent with NASEM recommendations and could minimise risk of bias that might allow the influence of corporate interests on the DGAC scientific report. Given that this report is the principal basis for the DGA, which are widely used in national and regional programmes as well as policies aiming to promote healthier diets in the USA, a more transparent DGA could be more trustworthy if its process included public disclosure of COI on the DGAC. Similar measures to disclose and manage COI among the USDA-HHS employees closely involved in the DGA process would further bolster public trust and confidence in the DGA.

## Supporting information

Mialon et al. supplementary material Mialon et al. supplementary material

## Supplementary material

For supplementary material accompanying this paper visit https://doi.org/10.1017/S1368980022000672. For the original data and replication files visit https://osf.io/f8uqn/.

## References

[1] USDA/HHS (2020) Who’s Involved – Dietary Guidelines for Americans. https://www.dietaryguidelines.gov/about-dietary-guidelines/process (accessed June 2021).

[2] USDA/HHS (2020) Purpose – Dietary Guidelines for Americans. https://www.dietaryguidelines.gov/about-dietary-guidelines/purpose-dietary-guidelines (accessed June 2021).

[3] United States 101st Congress (1990) National Nutrition Monitoring and Related Research Act of 1990 – Section 301 of PL 101–445 (7 USC 5341). https://www.congress.gov/bill/101st-congress/house-bill/160 (accessed June 2021).

[4] U.S. Department of Agriculture (2021) USDA Budget Explanatory Notes: Food and Nutrition Service. https://www.usda.gov/sites/default/files/documents/32fns2021notes.pdf (accessed June 2021).

[5] USDA/HHS (2020) History of the Dietary Guidelines – Dietary Guidelines for Americans. https://www.dietaryguidelines.gov/about-dietary-guidelines/history-dietary-guidelines (accessed June 2021).

[6] McMurry KY (2003) Setting dietary guidelines: the US process. J Am Diet Assoc 103, 10–16. 10.1016/j.jada.2003.09.031 14666494

[7] Nestle M (2013) Food Politics: How the Food Industry Influences Nutrition and Health. Berkeley: University of California Press.

[8] Corporate Accountability International (2020) Dietary Guidelines for Corporate America. https://www.corporateaccountability.org/wp-content/uploads/2020/06/Infographic-Dietary-Guidelines-Corporate-America.-pdf.pdf (accessed June 2021).

[9] Nestle M (2020) At Last: The 2020 Dietary Guidelines Advisory Committee. Food Politics. https://www.foodpolitics.com/2019/02/at-last-the-2020-dietary-guidelines-advisory-committee/ (accessed June 2021).

[10] USDA Office of Ethics (2021) Ethics Advisor’s Desk Reference – A Summary of Ethics Laws and Regulations for USDA Employees – Conflicting Financial Interests. https://www.ethics.usda.gov/rules/guides/deskref.htm#4_1 (accessed June 2021).

[11] Dietary Guidelines Advisory Committee (2020) Scientific Report of the 2020 Dietary Guidelines Advisory Committee: Advisory Report to the Secretary of Agriculture and the Secretary of Health and Human Services. Washington, DC: U.S. Department of Agriculture, Agricultural Research Service.

[12] U.S. Office of Government Ethics (2020) OGE Form 450. https://www.oge.gov/Web/OGE.nsf/OGEForms/072B8F6679028547852585B6005A2051/$FILE/OGEForm450Aug2020.pdf?Open (accessed June 2021).

[13] National Academies of Sciences, Engineering, and Medicine (2017) *Optimizing the Process for Establishing the Dietary Guidelines for Americans: The Selection Process*. Washington, DC: The National Academies Press; available at 10.17226/24637. 29240355

[14] International Committee of Medical Journal Editors (2021) Disclosure of Financial and Non-Financial Relationships and Activities, and Conflicts of Interest. http://www.icmje.org/recommendations/browse/roles-and-responsibilities/author-responsibilities--conflicts-of-interest.html (accessed June 2021).

[15] Institute of Medicine (2011) *Committee on Standards for Developing Trustworthy Clinical Practice Guidelines: Clinical Practice Guidelines We Can Trust*. Washington, DC: Institute of Medicine; available at https://www.ncbi.nlm.nih.gov/books/NBK209539/ (accessed June 2021).

[16] Lazarus D (2019) A Former Corn-Syrup Lobbyist is Drafting New Federal Dietary Rules (Seriously). https://www.latimes.com/business/lazarus/la-fi-lazarus-food-industry-shapes-dietary-guidelines-20190507-story.html (accessed June 2021).

[17] Waldman A & Armstrong D (2019) We Asked Public Universities for Their Professors’ Conflicts of Interest — and Got the Runaround. https://www.propublica.org/article/we-asked-public-universities-for-their-professors-conflicts-of-interest-and-got-the-runaround (accessed June 2021).

[18] Jackson D (2019) The Junk Food President Aims to Ruin American Nutrition. The American Prospect. https://prospect.org/power/junk-food-president-aims-ruin-american-nutrition/ (accessed June 2021).

[19] Hitt Nichols E (2020) First-Ever Birth-24 Month Dietary Guidelines: Deliberations and Complications. https://www.nutritioncoalition.us/2020-dietary-guidelines-info/birth-to-24-months-guidelines (accessed June 2021).

[20] Steele S , Ruskin G , Sarcevic L et al. (2019) Are industry-funded charities promoting “advocacy-led studies” or “evidence-based science”? A case study of the International Life Sciences Institute. Glob Health 15, 36. 10.1186/s12992-019-0478-6 PMC6545704 31155001

[21] Baker P , Melo T , Augusto Neves P et al. (2020) First-food systems transformations and the ultra-processing of infant and young child diets: the determinants, dynamics and consequences of the global rise in commercial milk formula consumption. Matern Child Nutr 17, e13097. 33145965 10.1111/mcn.13097 PMC7988871

[22] Fabbri A , Holland TJ & Bero LA (2018) Food industry sponsorship of academic research: investigating commercial bias in the research agenda. Public Health Nutr 21, 3422–3430. 30157979 10.1017/S1368980018002100 PMC10260999

[23] Bes-Rastrollo M , Schulze MB , Ruiz-Canela M et al. (2013) Financial conflicts of interest and reporting bias regarding the association between sugar-sweetened beverages and weight gain: a systematic review of systematic reviews. PLoS Med 10, e1001578. 24391479 10.1371/journal.pmed.1001578 PMC3876974

[24] Fabbri A , Lai A , Grundy Q et al. (2018) The influence of industry sponsorship on the research agenda: a scoping review. Am J Public Health 108, e9–e16. 10.2105/AJPH.2018.304677 PMC6187765 30252531

[25] Kotch A (2018) USDA Plagued with Conflicts as It Sets Dietary Guidelines. International Business Times. https://www.ibtimes.com/political-capital/usda-plagued-conflicts-it-sets-dietary-guidelines-2652024 (accessed June 2021).

[26] Jacobs A (2020) New Dietary Guidelines Draws Criticism from Health Advocates. https://www.nytimes.com/2020/06/17/health/diet-nutrition-guidelines.html (accessed June 2021).

[27] Moran G (2021) Questions Remain about Big Food’s Influence on the New Dietary Guidelines. Civil Eats. https://civileats.com/2021/01/28/questions-remain-about-big-foods-influence-on-the-new-dietary-guidelines/ (accessed August 2021).

