# Supplementary material for: Conflicts of interest for members of the US 2020 dietary guidelines advisory committee
Source: Public Health Nutr. 2022 Mar 21;27(1):e69. doi: 10.1017/S1368980022000672 (PMC10966930; doi:10.1017/S1368980022000672)
Supplement: Mialon et al. supplementary material [file S1368980022000672sup001.docx]

# Appendix 1: Bipartite network graph of COI between DGAC members and industry actors. Ties weighted by number ties over time.

[Figure A1 here]

Appendix 1 shows the whole network of links between researchers and industry actors for the entire period under study: in this bipartite weighted graph, the nodes are industry actors and researchers, and the ties denote a COI in a given year (to note that if the same COI is disclosed/recorded multiple times in the same year, it is only counted once). The thickness of the lines represent the strength of the relationship between DGAC members and industry actors (in this case, the number of years in which a COI is disclosed/recorded). The nodes are colored by partitions, which were found algorithmically using the Louvain method which leverages nodes’ connections to tease out “communities” in the network, and the ties are colored by the community of the source node. Here, communities are subgroups of nodes in the network that are typically well connected within the group and poorly connected outside the group.
